# Fungal Infections in Hospitalized Patients of Systemic Lupus Erythematosus: A United States Nationwide Cohort Analysis

**DOI:** 10.7759/cureus.65302

**Published:** 2024-07-24

**Authors:** Saman Tanveer, Chun-Wei Pan, Faria Sami, Maria E Romero Noboa, Diego M Cornejo Gonzalez, Kirtan Patolia, Fatima Tanveer, Daksh Ahluwalia

**Affiliations:** 1 Internal Medicine, John H. Stroger, Jr. Hospital of Cook County, Chicago, USA; 2 Internal Medicine, Allama Iqbal Medical College, Lahore, PAK; 3 Internal Medicine, CMH Lahore Medical College and Institute of Dentistry, Lahore, PAK

**Keywords:** clinical rheumatology, steroid use, nationwide inpatient sample (nis), fungal infectionss, systemic lupus erythromatosus

## Abstract

Introduction/objective

Immunosuppressive therapy is the cornerstone of management in patients with systemic lupus erythematosus (SLE). Patients on immunosuppressive therapy are at increased risk of developing opportunistic fungal infections. We conducted this analysis to describe the epidemiology, including incidence, risk factors, and outcomes, of fungal infections in hospitalized patients with SLE in the United States.

Method

A retrospective cohort study was performed by analyzing the National Inpatient Sample (NIS) 2016-2020 for all patients with a discharge diagnosis of SLE and fungal infections, including histoplasmosis, pneumocystosis, cryptococcosis, aspergillosis, and blastomycosis, as a primary or secondary diagnosis via ICD-10 (International Classification of Diseases 10th Revision) codes. Frequencies, demographics, and trends were determined and compared between hospitalized patients with SLE and those without SLE. STATA version 17 was used for data analysis. A p-value of ≤0.05 was considered statistically significant.

Results

In hospitalized SLE patients, there were lower odds of developing fungal infections in females (odds ratio (OR): 0.63 (95% confidence interval (CI): 0.49-0.80)) and higher odds in Hispanic (OR: 1.52 (95% CI: 1.16-1.98) and Asian (OR: 1.78 (95% CI: 1.15-2.75) populations. Steroid use (OR: 1.96 (95% CI: 1.58-2.42)), concomitant HIV infection(OR: 22.39 (95% CI: 16.06-31.22)), and the presence of leukemias (OR: 3.56 (95% CI: 1.67-7.59)) and lymphomas (OR: 3.29 (95% CI: 1.78-6.09)) in hospitalized SLE patients were significant predictors of fungal infection (p < 0.01). There were differences in the incidence of fungal infections based on geographical areas in the US, with blastomycosis being more common in the Midwest. From 2016 to 2020, there was a decline in the incidence rate of hospitalization per 100,000 for non-SLE patients with fungal infections (10.7 per 100,000 hospitalizations in 2016 versus 9.6 per 100,000 hospitalizations in 2020), whereas this rate remained steady for the SLE cohort (0.1 per 100,000 hospitalizations in 2016 versus 0.2 per 100,000 hospitalizations in 2020).

Conclusions

Hospitalized patients with SLE are at an increased risk of developing fungal infections, and this risk is increased further in patients who are males, are on steroid therapy, and have HIV or leukemia and lymphomas. Further studies can be done to explain the increased risk of fungal infections associated with these patient characteristics.

## Introduction

Systemic lupus erythematosus (SLE) is a multi-systemic autoimmune disorder that is characterized by the production of autoantibodies that cause tissue injury derived from chronic inflammation [1]. In the last 50 years, life expectancy in these patients has increased dramatically, in part due to the advancement of immunosuppressive agents, which have become the cornerstone of treatment [2]. However, the mortality rate remains considerably high when compared to the general population.

Infections represent one of the major culprits of morbidity and mortality in SLE, as patients exhibit an increased susceptibility, particularly for opportunistic infections. This is explained by the dysregulation of the immune system caused by the deposition of immune complexes and the infiltration of activated T cells into susceptible organs. Therapeutic immunosuppressive therapy contributes further to this phenomenon. More than 80% of SLE patients who require immunosuppressive agents for disease control use them for prolonged periods [3]. Specifically, the chronic use of corticosteroids increases the risk of infection by two- to 2.5-fold, and their concomitant use with cyclophosphamide has demonstrated a markedly increased rate of serious infections when compared to prednisone alone (45% versus 12%) [4-5]. Although used less frequently, belimumab and azathioprine have been classically linked with a lower risk of infections than other cytotoxic drugs or corticosteroids, except in patients with leukopenia [6-7].

Infections range widely from common presentations of typical microorganisms to opportunistic infections and atypical presentations that can mimic lupus flares, posing diagnostic and therapeutic challenges [8]. Even though approximately 80% of SLE infections are caused by bacteria, invasive fungal infections also occur, including *Candida*, *Aspergillus*, and *Cryptococcus*, and less commonly by *Histoplasma*, *Blastomyces*, and *Coccidioides*. *Pneumocystis jirovecii *is also accountable for severe pulmonary involvement and a higher severity and mortality rate in SLE patients, even in those not using immunosuppressants [9]. When compared to other patients on similar doses of these drugs, SLE patients present an increased predisposition to invasive fungal infections. Notably, disseminated fungal infections in this population can have a mortality risk as high as 50%, hence the importance of understanding the epidemiology of fungal infections in SLE [10]. To our knowledge, this is the first national database study that has examined information regarding this subject.

This article was previously presented as a poster at the ACR Convergence 2023 on November 10-15, 2023.

## Materials and methods

We performed a retrospective cohort study including all adult hospitalizations documented in the National Inpatient Sample (NIS) from 2016 to 2020 (available online at http://www.hcup-us.ahrq.gov). The NIS is the largest database of data from inpatient hospitalizations in the United States (US). It is part of the Healthcare Cost and Utilization Project (HCUP), which is maintained by the US Department of Health and Human Services. The NIS sample is stratified to represent all non-federal hospitals that provide acute care in the US. These hospitals are classified based on the rural/urban location, bed size, geographical localization, ownership, and teaching status. The NIS eventually includes a probability sample of about 20% from each stratum. All hospitalizations and, therefore, discharges are also documented and then weighted so they are nationally representative. Data from 47 statewide data organizations are included in the NIS 2016-2020 registry, and this includes about 97% of the US population. All principal and secondary diagnoses are recorded using International Classification of Disease, Tenth Revision, Clinical Modification (ICD-10-CM) codes. The principal diagnosis is defined as the main reason for hospitalization, and the secondary diagnoses are other active or inactive comorbidities the patients have. Institutional review board permission was not required as we used de-identified data from NIS for our study. According to HCUP policy, reporting of data between numbers 1 and 10 is not permitted, to protect privacy. 

Inclusion criteria and study variables

We identified all adult hospitalizations with a principal or secondary diagnosis of SLE in the NIS from 2016 to 2020 and further stratifying patients with the following concomitant diagnoses: aspergillosis, histoplasmosis, cryptococcosis, blastomycosis, or pneumocystosis. The ICD-10-CM codes for SLE and fungal infections can be found in the appendix. The hospitalizations of SLE patients were studied for detailed analyses and categorized into two groups, one with concomitant diagnoses of the aforementioned fungal infections and the other group without the fungal infections. We studied epidemiology and compared the two groups for demographics, socioeconomic, and disease characteristics. 

These patient characteristics were also compared between the five most common fungal infections diagnosed in hospitalized patients with SLE. In addition, trends in hospitalizations were compared between patients with and without SLE hospitalized with fungal infections.

Outcomes

Outcomes included inpatient mortality rates, healthcare burden assessed by total hospital charges and length of stay, and prevalence of complications, including sepsis, renal failure, end-stage renal disease (ESRD), posterior reversible encephalopathy syndrome, pulmonary embolism (PE)/deep venous thrombosis (DVT), failure to thrive, and healthcare variables, such as leaving against medical advice (AMA), transfer to another facility, and renal transplant.

Statistical analysis

STATA 17.0 (StataCorp, Texas, USA) was used to perform the analyses. Baseline characteristics were summarized using descriptive statistics and compared using Pearson’s χ2 and Wilcoxon rank-sum test as appropriate. Median and interquartile range (IQR) were used for non-normally distributed continuous variables. Univariable logistic regression analyses were used to calculate unadjusted odds ratios (ORs) for in-hospital death. All variables with P-values ≤ 0.2 were included in a multivariable logistic regression model. P values ≤ 0.05 were considered significant in the multivariable analysis. Wilcoxon rank-sum and Pearson’s χ2 test were done for other outcomes as appropriate.

## Results

In our study period between 2016 and 2020, there were a total of 886,820 adult hospitalizations (age > 18) with a primary or secondary diagnosis of SLE, of which 2,450 hospitalizations had a principal or a secondary diagnosis of a concomitant fungal infection based on the ICD-10 codes (see Table 1). Among these, 969 (39.5%) had aspergillosis, 480 (19.6%) had* Pneumocystis jirovecii*, 475 (19.4%) had cryptococcus (PJP), 465 (18.9%) had histoplasmosis, and 60 (2.4%) had blastomycosis infection. For each type of fungal infection, females were noted to be more frequent (88.6%, 86.0%, 67/4%, 76.0%, and 91.7%, respectively). However, males had higher odds of developing fungal infections. Hispanic patients and Asian and Pacific Islander patients with SLE who were hospitalized had a higher odds ratio (OR) of developing fungal infections. Patients in the group with the lowest annual median income (quartile = Q1) were noted to generally have more frequent histoplasmosis (35.5%), cryptococcal (34.7%), PJP (35.5%), and blastomycosis infections (50.0%), and aspergillosis was noted to be most prevalent in highest annual median income group (Q4), with 29.0% noted hospitalizations. Medicare was the most common insurance type for all infection groups (aspergillosis = 46.0%, histoplasmosis = 45.5%, cryptococcus = 40.0%, and blastomycosis = 81.8%) except private insurance for PJP infection hospitalizations (40.2%).

**Table 1:** Description of patient-related, hospital, and geographical characteristics of patients with systemic lupus erythematosus hospitalized with fungal infections.

	Aspergillosis	Histoplasmosis	Cryptococcosis	Pneumocystosis	Blastomycoses
N=	969	465	475	480	60
Mean age	51.37	46.54	47.74	50.94	60.58
Age categories					
18-44	30.93%	48.39%	42.11%	33.33%	16.67%
45-64	47.42%	37.63%	42.11%	50.00%	41.67%
65-84	21.13%	13.98%	15.79%	15.62%	41.67%
>85	0.52%	0.00%	0.00%	1.04%	0.00%
Sex					
Male	11.34%	13.98%	32.63%	23.96%	8.33%
Female	88.66%	86.02%	67.37%	76.04%	91.67%
Race					
White	50.00%	50.57%	38.04%	31.91%	41.67%
Black	23.44%	26.44%	21.74%	37.23%	50.00%
Hispanics	15.10%	20.69%	34.78%	17.02%	8.33%
Asian or Pacific Islanders	7.81%	1.15%	3.26%	7.45%	0.00%
Other	3.65%	1.15%	2.17%	6.38%	0.00%
Median household income					
1-43,999	25.39%	35.48%	34.74%	35.48%	50.00%
44,000-55,999	26.42%	31.18%	28.42%	21.51%	16.67%
56,000-73,999	19.17%	18.28%	20.00%	19.35%	16.67%
>74,000	29.02%	15.05%	16.84%	23.66%	16.67%
Charlson Comorbidity index					
1	9.79%	22.58%	14.74%	14.58%	16.67%
2	29.90%	17.20%	11.58%	14.58%	25.00%
3	60.31%	60.21%	73.68%	70.83%	58.33%
Insurance type					
Medicare	46.07%	45.45%	36.96%	33.70%	81.82%
Medicaid	17.80%	22.73%	32.61%	23.91%	9.09%
Private	34.03%	28.41%	28.26%	40.22%	0.00%
Uninsured	2.09%	3.41%	2.17%	2.17%	9.09%
Hospital bed size					
Small	19.59%	17.20%	3.16%	8.33%	16.67%
Medium	21.65%	16.13%	23.16%	32.29%	25.00%
Large	58.76%	66.67%	73.68%	59.38%	58.33%
Hospital region					
Northeast	13.40%	3.23%	20.00%	20.83%	8.33%
Midwest	20.10%	38.71%	16.84%	23.96%	58.33%
South	32.99%	41.94%	41.05%	38.54%	33.33%
West	33.51%	16.13%	22.11%	16.67%	0.00%
Hospital teaching status					
Rural	2.06%	8.60%	1.05%	2.08%	0.00%
Urban, nonteaching	13.92%	17.20%	10.53%	15.63%	8.33%
Urban, teaching	84.02%	74.19%	88.42%	82.29%	91.67%

Hospital and geographical characteristics

Most of the hospitalizations for all infection types were from large bed-sized hospitals (aspergillosis = 58.8%, histoplasmosis = 66.7%, cryptococcus = 73.7%, PJP = 59.4%, and blastomycosis = 58.3%). These hospitalizations were also the most frequent in urban teaching hospitals. There were also notable geographical differences in the distribution of fungal infection hospitalizations. Most aspergillosis infections were documented in the west (33.5%), histoplasmosis in the south (41.9%), cryptococcal infections in the south, PJP in the south (38.5%), and blastomycosis in the Midwest (58.3%). 

Secondary diagnoses and risk factors

**Figure 1:**
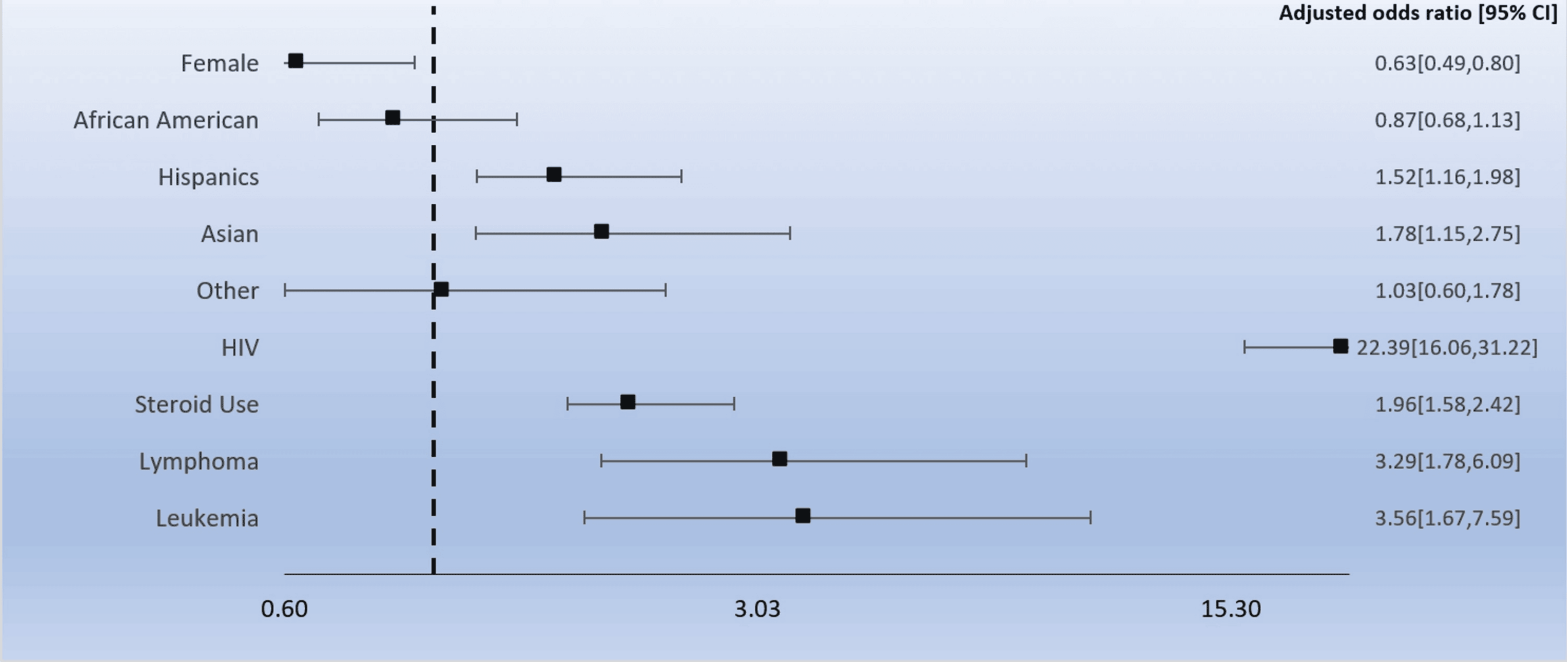
Predictors of fungal infections in hospitalized SLE patients SLE: systemic lupus erythematosus

Secondary diagnoses of HIV status, chronic steroid use, lymphoma, and leukemia were also analyzed for each fungal infection. Frequent use of chronic steroids was noted in each subgroup (aspergillosis = 27.8%, histoplasmosis = 20.4%, cryptococcus = 27.4%, PJP = 33.3%, and blastomycosis = 8.3%). Positive HIV status was noted in all subgroups with the following proportions: aspergillosis group = 5.7%, histoplasmosis = 6.5%, cryptococcus = 10.5%, PJP = 21.9%, and blastomycosis = 8.3%. Lymphoma and leukemia diagnoses were relatively low. Multivariate regression analysis was done to adjust for confounders and identify the odds of fungal infection for each secondary diagnosis as discussed. HIV-positive status (OR 22.4, p < 0.01), chronic steroid use (OR 2.0, p < 0.01), lymphoma (OR 3.3, p < 0.01), and leukemia (OR 3.6, p < 0.01) were all found to be significantly associated with higher odds for fungal infections (Figure 1).

Morbidity and mortality

The highest mortality was noted for PJP hospitalizations (17.7%), followed by blastomycosis infections (16.7%), aspergillosis (11.3%), cryptococcal infections (8.4%), and lastly histoplasmosis (2.2%).

We also observed a higher number of comorbidities in SLE patients hospitalized with fungal infections compared to those without fungal infections, with most patients having three or more comorbidities as indicated by the Charleson Comorbidity Index (CCI) of 3 or higher.

Trends of fungal infection-related hospitalizations

**Figure 2:**
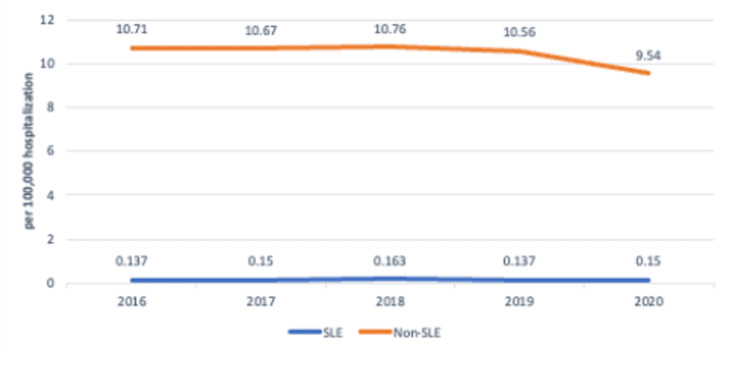
Trend of fungal hospitalization in SLE versus non-SLE patients over the years SLE: systemic lupus erythematosus

When analyzed from 2016 to 2020, there was a decline in the rate of hospitalizations per 100,000 for non-SLE patients with fungal infections (10.7 per 100,000 hospitalizations in 2016 versus 9.6 per 100,000 hospitalizations in 2020) (see Figure 2). This rate remained steady for the SLE cohort (0.1 per 100, 000 hospitalizations versus 0.2 per 100,000 hospitalizations), but the difference was not statistically significant.

## Discussion

We analyzed the NIS from 2016 to 2020 for patients with SLE and fungal infections, including histoplasmosis, pneumocystis, cryptococcosis, aspergillosis, and blastomycosis. Overall, patients with SLE have a higher risk of developing fungal infections. The rates of invasive fungal infections are highest in the first three years following the diagnosis of SLE [11]. Patients with SLE are more susceptible to infections due to immune dysregulation with innate and adaptive systems defects [2]. The impaired phagocytic cell activity and impaired T-cell activity predispose to fungal infections [12]. Immunosuppressive agents also have a substantial role in the risk of infections. This, in turn, is the major source of morbidity and mortality in this population [2]. In the cohort by Hsu et al., the incidence rate of opportunistic infections in SLE is 43.1/1000 person-years [13]. Mortality can be four times more frequent in SLE patients with invasive fungal infections [14].

This study shows that the risk of fungal infections is higher in males with SLE. This finding is in line with prior literature; males with SLE tend to have more complications and more severe disease than females [15]. This finding was also observed in the nationwide cohort by Feldman et al., where they showed a significantly increased risk of infection among males with SLE [16]. It was also noted that Hispanic and Asian populations with SLE have a higher risk of fungal infections. A study by Alza et al. revealed a 7.5% prevalence of fungal infections in the Colombian population, with invasive candidiasis as the most common infection [17]. Similarly, a Taiwanese series by Chen et al. showed the highest incidence of cryptococcosis [18]. Meanwhile, Feldman et al. showed higher infection rates in African American and Native American SLE patients. These patients, in turn, also had a higher incidence of lupus nephritis [16].

Some factors are predictors of fungal infections in patients with SLE, including steroid use, HIV infection, and hematologic malignancies like leukemia and lymphoma. It is known that glucocorticoids or other immunosuppressants contribute to immune dysregulation in SLE patients, increasing the susceptibility to invasive fungal infections. Lymphopenia and accumulated glucocorticoid use, in particular, are associated with invasive fungal infections in lupus patients [19]. Steroids suppress cell-mediated immunity and reduce monocytes and macrophages, increasing the risk of opportunistic infections with higher doses related to a higher risk of infection [2]. There is a positive correlation between the cumulative dose of steroids and infection, as seen in the cohort by Banerjee et al. [20]. In the cohort by Yang et al., they confirmed that the risk of opportunistic infection is 136-fold higher in SLE patients treated with steroids in the first three months and that medium to high doses increased the risk [21]. HIV and SLE have similar immune abnormalities with a general decrease of CD4+ T cells, leading to an increased risk for opportunistic infections [22]. High mortality rates in cases of invasive aspergillosis are seen in the presence of certain risk factors, including high daily steroid dose (more or equal to 20 mg of prednisone), recent pulse steroid therapy, use of immunosuppressants, and CMV viremia [23]. Hematologic malignancies as predictors for fungal infection in SLE patients were also noted in the cohort by Su et al. [11]. Prior literature has shown that hematological malignancies pose a high risk for fungal infections given evolving treatments, allogeneic stem-cell transplantation, performance status, comorbidities, and environmental exposure [24,25].

Our study reveals that from 2016 to 2020, the incidence of SLE patients with fungal infections has remained steady, while it has declined in non-SLE patients with fungal infections; however, this difference was not statistically significant. A study done in Western Australia also revealed the hospitalization rates for opportunistic infections in SLE patients did not change significantly from 1985 to 2015. However, it was seen that mycobacterial and pneumocystis infections decreased while viral and mycotic infections increased [26].

To plan for prophylaxis in SLE patients, it is essential to assess a patient's risk factors for infections before starting immunosuppressive agents. There are few guidelines on preventing infections in SLE but not much on opportunistic infections. Guidelines include routine immunization against pneumococcus, influenza, HPV, and herpes zoster, as well as screening for hepatitis B and C virus, tuberculosis, and strongyloidiasis depending on the country of origin and travel history. Moreover, pneumocystis prophylaxis may be considered in patients on 16-20 mg of prednisone daily for at least eight weeks [12]. On the other hand, in the review by Kapoor et al., there was a low prevalence of pneumocystis pneumonia in patients with SLE; hence, prophylaxis with Bactrim is not sustained unless there is concurrent HIV/AIDS [27].

Despite its strengths, studies relying on the NIS have certain limitations. The NIS database relies on ICD-10 coding to identify diagnoses, instead of clinical assessment. The study, therefore, relies on the accuracy of coding; human error cannot be completely excluded from the process of billing and coding. Data about management, treatment, and distinct laboratory findings cannot be obtained from the registry, which could have helped assess some variables better by excluding their confounding effect. Since the NIS documents the number of hospitalizations only, repetitive hospitalization for the same patients could not be identified or excluded. The NIS findings can only apply to the inpatient population, and findings do not reflect clinic patients.

## Conclusions

Our study is the first to analyze the demographic, socioeconomic, and disease characteristics of SLE patients hospitalized with fungal infections. The study has not only shed light on the types of fungal infections, which usually affect hospitalized SLE patients, but also demonstrated various patient-specific factors that can increase or decrease the odds of developing these fungal infections. Studies like these can pave the way for further research to better establish these characteristics as actual risk factors for fungal infections and look for ways to prevent or minimize these infections.

## Appendices

**Table 2:** ICD-10-CM codes for systemic lupus erythematosus and fungal infections ICD: International Classification of Disease, CM: clinical modification

ICD-10-CM codes	Disease
Systemic lupus erythematosus	M32
Aspergillosis	B44.0, B44.1, B44.2, B44.7, B44.81, B44.89, B44.9
Histoplamsosis	B39.0, B39.1, B39.2, B39.3, B39.4, B39.5, B39.9
Cryptococcosis	B45.0, B45.1, B45.2, B45.3, B45.7, B45.8, B45.9
Blastomycosis	B40.0, B40.1, B40.2, B40.3, B40.7, B40.81, B40.89, B40.9
Pneumocystosis	B59
